# Supplementary material for: The Therapeutic Potential of ADAMTS8 in Lung Adenocarcinoma without Targetable Therapy
Source: J Pers Med. 2022 May 30;12(6):902. doi: 10.3390/jpm12060902 (PMC9225423; doi:10.3390/jpm12060902)
Supplement: Supplementary file 1 [file jpm-12-00902-s001.zip › jpm-1695511-supplementary.pdf]

Supplementary file

Supplementary Figure S1. The expression of ADAMTS family of lung adenocarcinoma in our cohort. The expression levels of the aggrecanases or proteoglycanases (A), the procollagen N-propeptidases (B), the cartilage oligometric matrix protein-cleaving enzymes (C), the von-Willebrand Factor proteinase (D) and the Orphan enzymes (E) of in-house cohort. ns, not significant.

Supplementary Figure S2. The expression of ADAMTS family of lung adenocarcinoma in TCGA cohort. The expression levels of the aggrecanases or proteoglycanases (A), the procollagen N-propeptidases (B), the cartilage oligometric matrix protein-cleaving enzymes (C), the von-Willebrand Factor proteinase (D) and the Orphan enzymes (E) of TCGA cohort. ns, not significant.

Supplementary Figure S3. The predicted miRs of ADAMTS8. The correlation of ADAMTS8 and ten miRs.

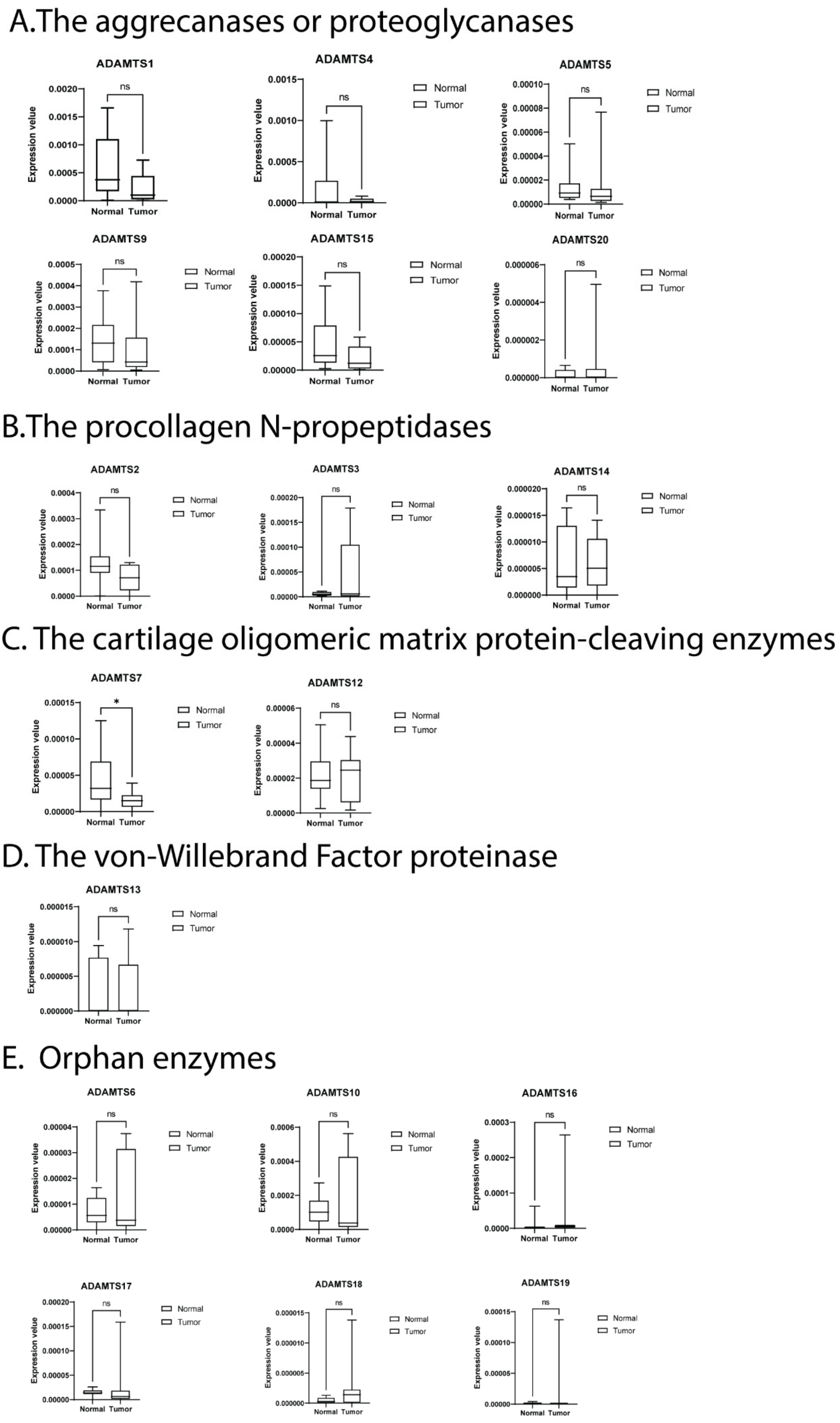
A.The aggrecanases or proteoglycanases
ADAMTS1
ns
ADAMTS4
ns
ADAMTS5
ns
ADAMTS9
ns
ADAMTS15
ns
ADAMTS20
ns
B.The procollagen N-propeptidases
ADAMTS2
ns
ADAMTS3
ns
ADAMTS14
ns
C. The cartilage oligomeric matrix protein-cleaving enzymes
ADAMTS7
*
ADAMTS12
ns
D. The von-Willebrand Factor proteinase
ADAMTS13
ns
E. Orphan enzymes
ADAMTS6
ns
ADAMTS10
ns
ADAMTS16
ns
ADAMTS17
ns
ADAMTS18
ns
ADAMTS19
ns
Expression velue
Normal
Tumor

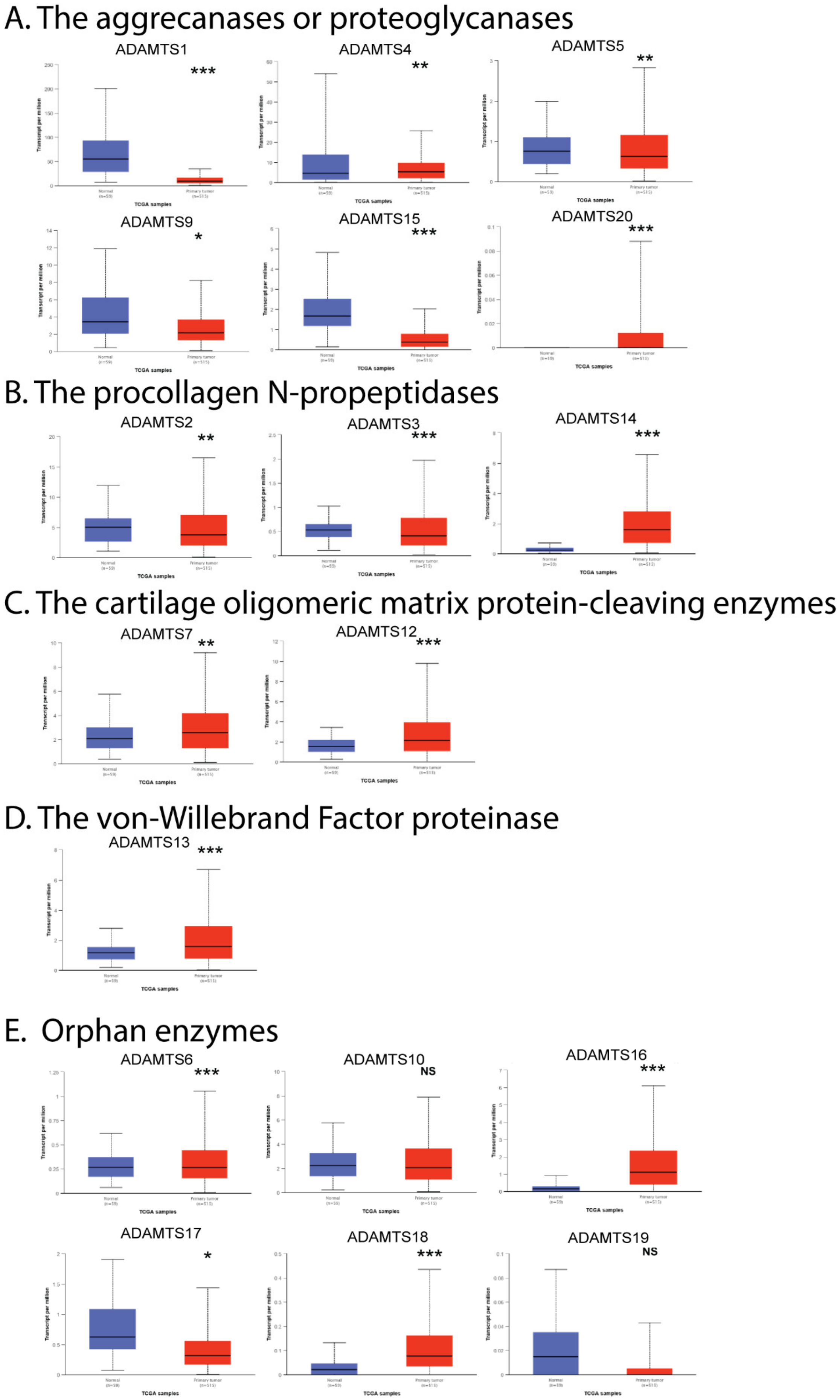
A. The aggrecanases or proteoglycanases
ADAMTS1
***
ADAMTS4
**
ADAMTS5
**
ADAMTS9
*
ADAMTS15
***
ADAMTS20
***
B. The procollagen N-propeptidases
ADAMTS2
**
ADAMTS3
***
ADAMTS14
***
C. The cartilage oligomeric matrix protein-cleaving enzymes
ADAMTS7
**
ADAMTS12
***
D. The von-Willebrand Factor proteinase
ADAMTS13
***
E. Orphan enzymes
ADAMTS6
***
ADAMTS10
NS
ADAMTS16
***
ADAMTS17
*
ADAMTS18
***
ADAMTS19
NS

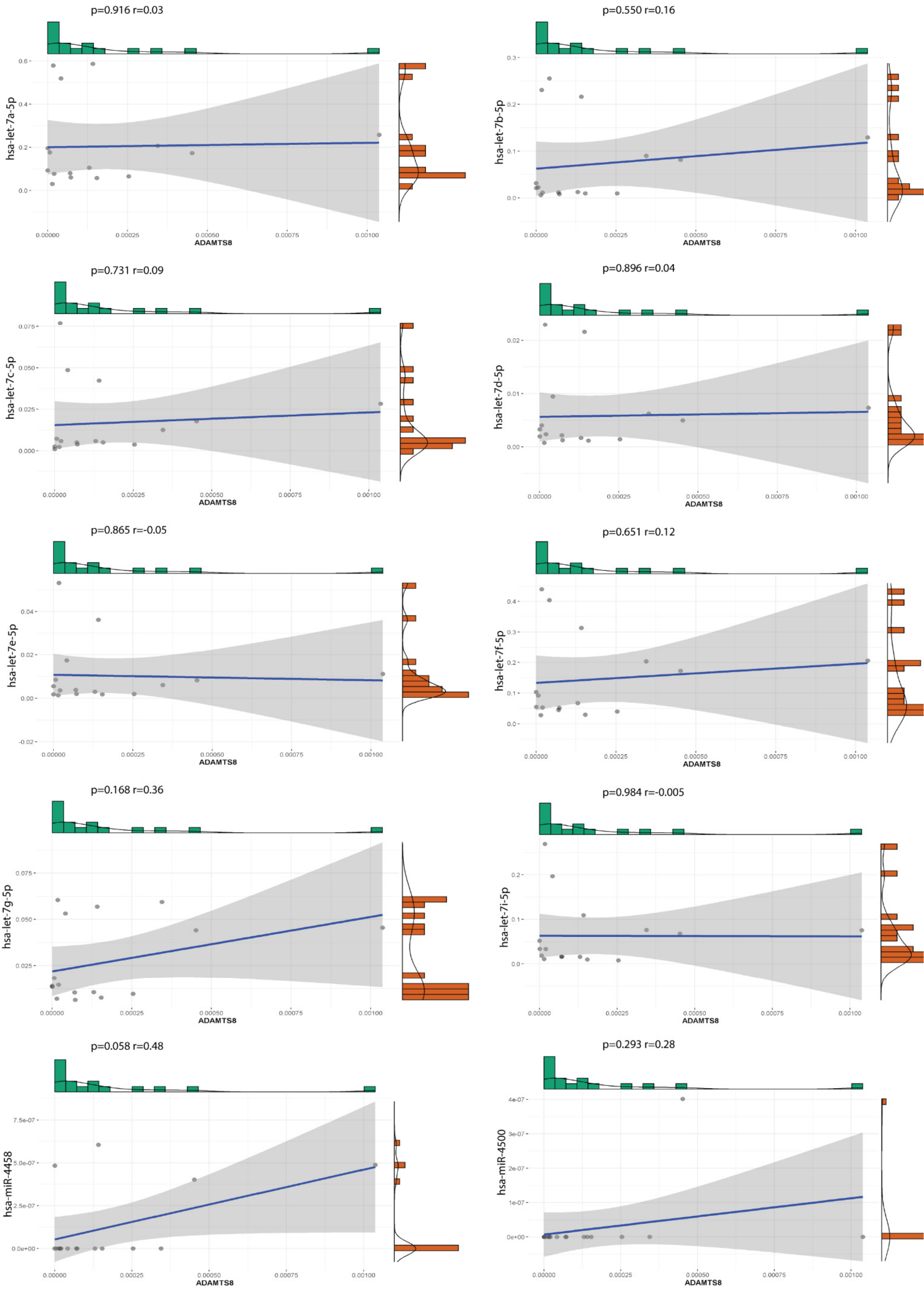
p=0.916 r=0.03
hsa-let-7a-5p
ADAMTS8
p=0.550 r=0.16
hsa-let-7b-5p
ADAMTS8
p=0.731 r=0.09
hsa-let-7c-5p
ADAMTS8
p=0.896 r=0.04
hsa-let-7d-5p
ADAMTS8
p=0.865 r=-0.05
hsa-let-7e-5p
ADAMTS8
p=0.651 r=0.12
hsa-let-7f-5p
ADAMTS8
p=0.168 r=0.36
hsa-let-7g-5p
ADAMTS8
p=0.984 r=-0.005
hsa-let-7i-5p
ADAMTS8
p=0.058 r=0.48
hsa-miR-4458
ADAMTS8
p=0.293 r=0.28
hsa-miR-4500
ADAMTS8
